# Molecular characterization of *Gyps africanus* (African white-backed vulture) organic anion transporter 1 and 2 expressed in the kidney

**DOI:** 10.1371/journal.pone.0250408

**Published:** 2021-05-04

**Authors:** Bono Nethathe, Rephima Phaswane, Aron Abera, Vinny Naidoo

**Affiliations:** 1 Department of Paraclinical Sciences, Faculty of Veterinary Science, University of Pretoria, Pretoria, South Africa; 2 Department of Food Science and Technology, School of Agriculture, University of Venda, Limpopo, South Africa; 3 Department of Pathology, Faculty of Veterinary Science, University of Pretoria, Pretoria, South Africa; 4 Inqaba Biotechnology, Pretoria, South Africa; University College Dublin, IRELAND

## Abstract

Gyps species have been previously shown to be highly sensitive to the toxic effects of diclofenac, when present in their food sources as drug residues following use as a veterinary medicine. Vultures exposed to diclofenac soon become depressed and die with signs of severe visceral gout and renal damage on necropsy. The molecular mechanism behind toxicity and renal excretion of uric acid is still poorly understood. With the clinical pictures suggesting renal uric acid excretion as the target site for toxicity, as a first step the following study was undertaken to determine the uric acid excretory pathways present in the African white-backed vulture (*Gyps africanus*) (AWB), one of the species susceptible to toxicity. Using transcriptome analysis, immunohistochemistry and functional predictions, we demonstrated that AWB makes use of the *organic anion transporter 2* (*OAT2*) for their uric acid excretion. RT-qPCR analysis subsequently demonstrated relatively similar expression of the *OAT2* transporter in the vulture and chicken. Lastly docking analysis, predicted that the non-steroidal drugs induce their toxicity through an allosteric binding.

## 1. Introduction

Diclofenac related toxicity and mass die offs have been well documented for three *Gyps* species on the Asian continent following contamination of their food source from the veterinary use of the drug in cattle [1]. Despite the birds dying as early as 48 hours post exposure with an unprecedented population mortality in excess of 99% with millions of birds being found dead, the mechanism behind toxicity remains poorly understood [2]. Current speculation is that toxicity is linked to renal functionality as a result of pathological finding of visceral gout in recently dead birds. Gout is the pathological finding that results from the precipitation of urate salts into the abdominal cavity once the saturation point of plasma has been reached. As a substance uric acid is produced by the liver from the breakdown of purines both endogenous and exogenous, and maintained as a finely controlled suspension in the plasma [3]. To further maintain the latter homeostasis, the kidneys play a key role in excreting uric acid evident as the white residue in avian excreta. Despite the importance of the renal system in uric acid excretion, the uric acid excretory pathway in vultures in yet to be described. Nonetheless it has been speculated that inhibition of renal functionality likely explains the development of gout.

From studies in humans (low uric acid producers), it is known that the kidneys contribute to the maintenance of homeostasis by removing uric acid through a combination of glomerular filtration and by proximal convoluted tubules (PCT) secretion [4]. For the former, the process is facilitated by differential pressure across the glomerulus in a size dependent manner while the latter is mediated by an active transport mechanism. Of the two the PCT is the more complex physiological process as the cells herein are a major physiological barrier to the passive diffusion of charged hydrophilic molecules from the blood into the tubules. To overcome this, the cells make use of dozens of membrane-bound transporter proteins; the solute carriers (SLC) transport proteins identified as *OAT1* to *OAT4* (*SLC22A6* to *SLC22A8* for *OAT1* to *OAT3* and *SLC22A11* for *OAT4*) [5–8]; the *multidrug resistance protein* (*MRP2*/*ABCC2* and *MRP4*/*ABCC4*) and the *urate transporter 1* (*URAT1*) [9, 10]. The *OAT1* and *OAT3* transporters are localized in the basolateral membrane of renal proximal tubule cells and mediate the movement of organic anions such as uric acid from the plasma into the PCT cells through dicarboxylate/organic anion exchange. At the same time a sodium-dependent dicarboxylate (NADC-3) co-transporter facilitates the transfer of the excreted dicarboxylate back into the cells. From within the cellular cytoplasm uric acid is secreted by the *MRP2* and *MRP4* transporters located on the apical cell membrane of the renal tubule into the renal tubular. The process of the OAT and MRP transporter is known as tubular secretion. At the same time, uric acid present within the tubule, either from glomerular filtration or from tubular secretion, can be reabsorbed back into the systemic circulation via dicarboxylate or hydroxyl ions and monocarboxylate exchange by *OAT4* and *URAT1* respectively which are also localized in the apical membrane in the process known as tubular reabsorption [7, 11, 12].

Not surprisingly with this complex mechanism, uric acid homeostasis is based on a fine balance between glomerular filtration, tubular secretion and tubular reabsorption. This fine balance is subject to the effect of any substance that influences uric acid formation, or substances that interfere with excretion. For the former a high protein diet can result in an increase in uric acid formation, while drug substances can inhibit the functionality of the transport proteins. In addition to the natural anionic substances, the OATs are also involved with the excretion of a number of drugs such as the nonsteroidal anti-inflammatory drugs (NSAIDs), antiviral drugs, and β-lactam antibiotics [13]. With the interaction of drugs with the transporters, studies have also revealed that probenecid and other uricosuric drug like the NSAIDs in the process of being excreted also inhibits OAT mediated transport of many organic anions [14] making them useful for the treatment in gout in people.

While the mechanism of excretion in the vulture is unknown, from limited information in the chicken, the excretion of uric acid follows a similar mechanism as in humans with some key differences [15]. Unlike in mammals which rely mainly on urea as their nitrogenous excretory product, birds utilise predominantly uric acid for excretion i.e. birds are uricotelic and produce substantially higher concentrations of uric acid [15–17]. As a result, the excretory needs for uric acid in birds far exceeds glomerular filtration capacity with the result that the tubules are responsible for up to 80% of the excretion of uric acid [18]. To further enhance uric acid excretion, birds do not reabsorb uric acid and as yet no *URAT1* transporters have been described. From studies by [15] using primers derived from human OAT transport sequence, the chicken basal uric acid excretory pathway has been described to be mediated by the *OAT1* and *OAT3* transporter. With no information being available for the old world vultures, for this study, we attempt to characterise the uric acid transporter proteins as the first step in establishing the mechanism of diclofenac toxicity in the vulture. We also make use of the chicken as our comparator species, as it is the only non-vulture species demonstrated to be sensitive to the toxic effects of diclofenac under laboratory conditions albeit at a 10 fold higher dose of 10 mg/kg [2].

## 2. Materials and methods

### 2.1. Identification of the transporters present in the vulture

#### 2.1.1. OATs studies on AWB vulture and domestic chicken kidneys using published primers

Before the commencement of experiments, ethical clearance for collection of samples was conducted according to the guidelines and regulations approved by University of Pretoria Animal Ethics committee (AEC) (project number: V108-16). The uric acid transporters were evaluated in two adult AWB (*Gyps africanus*) that were euthanised for medical reasons. For controls when necessary chicken or mouse renal tissue was used. For AWBV1 and a chicken, total RNA was extracted from kidney samples using the RNeasy plus mini kit (Qiagen) according to the manufacturer’s instructions and stored at -80°C for reverse transcription. Reverse transcription was conducted using ClontechSmart MMLV Reverse Transciptase kit according to the manufacture`s instruction. *OAT3* chicken primers (forward 5`CCCTTCTTCCTCTTCTTCCTCG-3`and reverse 5`-TGGATCAGATAAATGCTGACCCC-3`) described by [15] were used for the partial amplification (Primer supplied by Integrated DNA Technologies, South Africa). PCR reactions were prepared using Fermentors kit following manufacturer’s instructions. The amplification conditions were as follows: 38 cycles with a denaturing temperature of 94°C, an annealing temperature 61.6°C and an elongating temperature of 72°C [15]. Amplicons were sequenced using an ABI 3500X1 genetic analyser (Inqaba Biotechnology, South Africa). Alignment comparisons were made with sequence (BBSRC Chick EST ID 603812145F1) described by [15]. The obtained sequence was also further analysed using blastn algorithm on National Centre of Biotechnology Information (NCBI) [19].

#### 2.1.2. OATs transcriptome analysis in AWB vulture

For Next Generation Sequencing, total RNA was extracted from the above AWB vulture kidney and sequence at Agricultural Research Council (ARC), (Onderstepoort, Pretoria, South Africa). cDNAs were sequenced using Illuminia Truseq mRNA stranded Ran preparation kit on Hiseq 2500 v4 2x125bp chemistry model. The result obtained from sequencing was 50 million short reads which were 125 nucleotides long each. Subsequent analysis was undertaken on the Galaxy platform using FASTQC [20], after which the adapters were removed using Trimmomatic [21]. The pre-processed reads were assembled into transcripts using TRINITY [22]. The assembled transcriptome was converted to a local Blast database and *OAT1* and *OAT2* sequences were identified based on the predicted sequences from the Golden Eagle *OAT1* (XM_011601043.1) and *OAT2* (XM_011585794.1). The Golden Eagle was selected since the two species have been shown to be closely related [23].

#### 2.1.3. Confirmation of AWB transcriptome *OAT1* and *OAT2* genes using Sanger sequencing

Fresh total RNA was extracted from the second AWB vulture kidney tissue, to prevent the effect of storage, using Quick RNA Miniprep kit from Zymo Research (USA). cDNA synthesis was performed using Lunascript RT supermix kit from New England Biolabs (NEB), (USA). Primers for *OAT1* and *OAT2* were designed based on the transcriptome sequences: OAT1(F): GACCTTGTCTGCAGCTACCG; OAT1(R): CCAGAGCTGCTTTATTCCTCCAAG; OAT2(F): CTCATGTTGCTGCTCCTTAGTACA and OAT2(R): CTAGGTGGACAGTAAAGGCTCTTT. PCR was performed using One taq polymerase kit from NEB. The amplification protocol was as follows: Initial denature 94°C for 30 sec, 40 cycles of (denature 94 ^0^C for 30 sec; annealing 55 ^0^C for 30 sec and elongation 68 ^0^C for 2 min) and final elongation 68°C for 5 min. The purified PCR products was sequenced using an ABI 3500X1 genetic analyser.

#### 2.1.4. Phylogenetic analysis

The obtained forward and reverse OATs Sanger sequences of AWB vulture, were aligned using Clustal Omega [24] to obtain consensus sequences. For the construction of the phylogenetic tree, 26 (*OAT1*) and 39 (*OAT2*) closely related avian species with more than 85% similarities with the AWB vulture *OAT1* and *OAT2* were retrieved from Genbank. The avian species downloaded from Genbank (NCBI) belongs to the following orders: Papaeognathae, Galoanseres and majority of the species were Neoaves. The downloaded taxa were aligned using muscle algorithm (Molecular Evolutionary Genetics Analysis version X (MEGA X) software package [25, 26]. After alignments, the gaps were classified as missing data. The genetic distance and the statistics of the nucleotide composition of all the taxa were computed in MEGA X version. The phylogenetic relationships were constructed using maximum likelihood (ML) estimate of Tamura-Nei model and General Time Reversible model [27, 28]. To assess nodal reliability, bootstrap analysis was conducted with 1000 replicates for the phylogenetic tree topologies [29]. The accession numbers of OATs (1 and 2) from different avian species included in the phylogenetic tree are listed in (S1 Table).

### 2.2. Predictive protein functionality

#### 2.2.1. Protein locality

Prior to incubation with the primary antibodies sequence analysis was used to first show similarity between the polyclonal rabbit anti-OAT3 antibody (Whitehead scientific, South Africa) binding site and AWB OAT1, corresponding to immunogen sequence KKEEGERLSL EELKLNLQKE ISLAKAKYTA SDLFRIPMLR at 72% similarity. Three day old female CD1 mouse kidney (n = 5) was used as the control since *OAT1* sequences are not recorded in the chicken. The kidneys were preserved by immersion fixation. The method previously described by [30] was employed with some modifications. The kidneys were briefly perfused with phosphate buffered saline (PBS) at an osmolality of 298 mOsm/kg H2O (pH 7.4) to remove all blood, followed by perfusion with a periodate-lysine-2% paraformaldehyde (PLP) solution for 10 min. After perfusion, the kidneys were removed and sliced into sections (1–2 mm thick), which were further fixed by immersion in the same fixative overnight at 4°C. After fixation, kidneys were embedded in wax and cut transversely at a thickness of 4 μm using a microtome. Sections were processed for immunohistochemistry using an indirect immune peroxidase method. All sections were washed three times in PBS containing 50 mM NH4Cl for 15 min. The sections were first treated with a graded series of ethanol, and then incubated for 4 h with solution A (PBS containing 1% bovine serum albumin (BSA), 0.05% saponin, and 0.2% gelatin). The tissue sections were thereafter incubated overnight at 4°C with the antibodies (1:3000) diluted in solution A.

After several washes in solution B (PBS containing 0.1% BSA, 0.05% saponin, and 0.2% gelatin), the tissue sections were incubated for 2 h in avidin-biotin-peroxidase-conjugated Goat anti-rabbit IgG (H+L) Fab fragment (White head Scientific, South Africa) diluted 1:100 in solution C (PBS containing 1% BSA). The samples were then rinsed in solution B, and then in 0.05 M Tris buffer (pH 7.6). To detect avidin-biotin-peroxidase, the sections were incubated in 0.1% 3,3’diaminobenzidine (DAB, Sigma) in 0.05 M Tris buffer for 5 min; H_2_O_2_ was added to a final concentration of 0.01% and the incubation continued for 10 min. The sections were washed three times with 0.05 M Tris buffer, dehydrated with a graded series of ethanol. All samples were examined with a light microscope.

#### 2.2.2. Protein prediction analysis

The obtained *OAT1* and *OAT2* sequences were converted by Expasy to protein sequences and the open reading frame ascertained. After the deduced amino acids sequences were analysed by following softwares for transmembrane helix prediction using TMHMM [31], and PHYRE2 (Protein homology recognition engine) with Scan Prosite [32] for prediction of 3-D structures and/or functionality. The *N*-glycosylation sites were predicted using PROTTER sequence database [33] and other possible post-glycosylation and phosphorylation sites were investigated with Expasy database studies. The OAT transporters were also analysed using TrSSP (The Transporter Substrate Specificity Prediction Server) [34] to ascertain if the predicted protein was likely an anion transporter.

#### 2.2.3. Expression of organic anion transporter 2

For *OAT2*, reverse transcriptase (RT) -qPCR was performed on AWB vulture and chicken cDNA (template) targeting GAPDH (housekeeping gene) [35] and for negative control no template was added, using specific primers: chickenOAT2_F: ACCATCTCCACTGAGTGGGAC, chickenOAT2_R: CGGCCGAACCTGTCTGAAAG and vultureOAT2_F: CATCTCCACGCAGTGGGAC, vultureOAT2_R: CGTCCGAACCTGTCTGAAAGG. All reactions were performed on CFX96 Real-Time PCR Detection System. The thermal cycling conditions for the RT-qPCR were as follows: 1 cycle at 95°C for 60 sec, 40 cycles of amplification at 95°C for 15 sec and annealing at 60°C for 30 sec. The average of the quantification cycle (Cq) was determined using manual quantification settings and was normalized using GAPDH Cq values. The formula used was as follows: normalized OAT2 Cq = OAT2 Cq—GAPDH Cq [35].

#### 2.2.4. Protein docking evaluations

Based on the result of the transporter specificity and *N*-glycosylation, predicted drug binding to *OAT2* was undertaken. The major pocket was evaluated by fPocket via the PHYRE2 platform, since large pockets are generally considered binding sites. The specific binding affinities of diclofenac and urate to the chicken, vulture or human *OAT2* transporters were evaluated at the molecular level in Swissdock using template structure generated by PHYRE2 and run on automatic settings. The resultant ΔG between urate and diclofenac and other NSAIDs (meloxicam, ketoprofen, carprofen, nimesulide, and tolfenamic acid) were obtained. With the point of binding of diclofenac not known, its binding site was taken as that point with the highest affinity diclofenac or uric acid. When their respective strongest affinities differed, the ΔG for the overlapping binding was determined by using diclofenac binding site as the likely point of attachment.

## 3. Results

### 3.1. Identification of the OAT transporters present in the AWB vulture using published primers, Next generation and Sanger sequencing

Poor amplification was achieved in the AWB vulture using the *OAT3* primers published by Duda et al. (2005) [15]. In contrast for the chicken, the generated sequence aligned with the published *OAT3*-like partial sequence (BBSRC Chick EST ID 603812145F1, 556bp) and revealed 97.11% similarity, indicating that the primers amplified the correct segment. On Blast analysis, the generated chicken sequence was however only similar to the *OAT1* sequence of other avian species with a highest similarity of 83% with the *Pelecanus crispus* (dalmatian pelican) and 81.4% for the *Aquila chrysaetos* (golden eagle) with no similarity evident with any published chicken sequence. Further no chicken *OAT3* partial sequence was present in the NCBI database. Moreover the *OAT3* transporter, as far as we could ascertain, has not been described in any bird species in the NCBI database. This leads us to speculate that the primers published by [15] rather amplified *OAT1*. In subsequent analysis focus was placed on only *OAT1* and *OAT2*.

**Fig 1:**
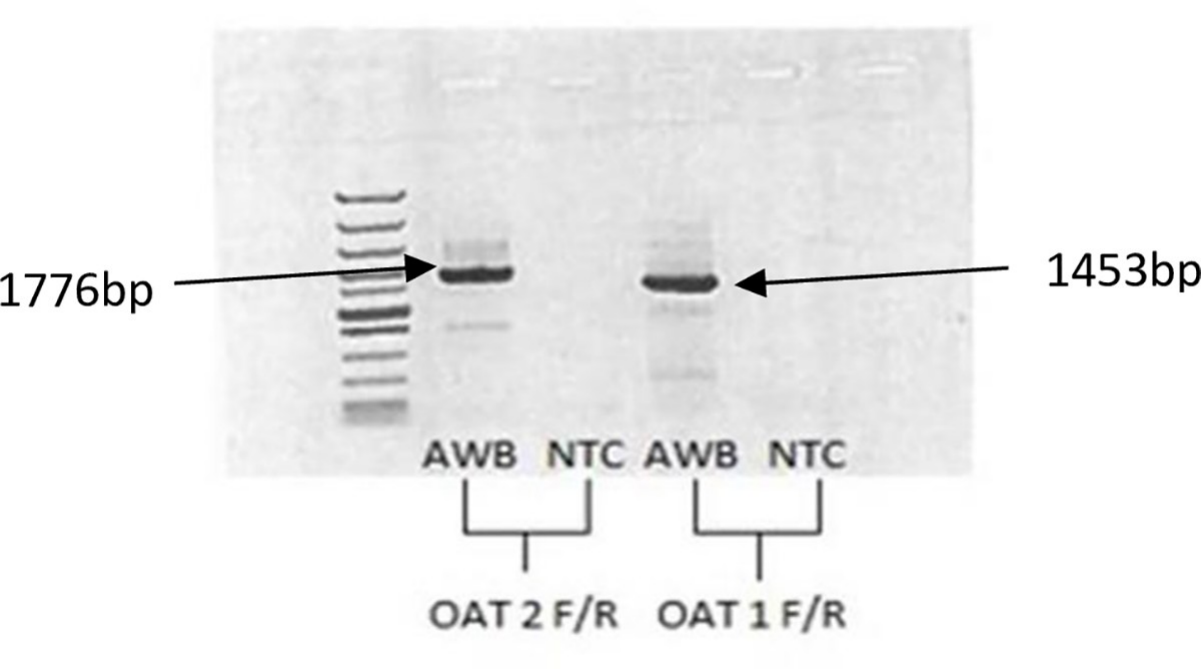
Conventional PCR amplified OAT1 and OAT2 gene from AWB vulture`s kidney, no product was obtained when the template was omitted, molecular size (100bp) is indicated on the left. NTC = no template control.

As a next step in analysis we reverted to full transcriptome analysis. Using extracted total RNA and next generation sequencing, the raw reads (PRJNA560189) of the renal sample were assembled in Trinity. Using the golden eagle predicted sequences for *OAT1* and *OAT2* as reference, the AWB sequences aligned with the former with similarity of 98.89 and 98.07% respectively (*OAT1* -MN691106; *OAT2* -MN691107). These sequences were subsequently confirmed using Sanger sequencing (Fig 1), with good similarity for *OAT1* (MK854995) and *OAT2* (MK879652) at 98.81 and 99.34% respectively. Sanger sequence similarity with chicken *OAT2* was at 88.05% (*OAT1* has not as yet been identified in the chicken).

### 3.2. Phylogenetic analysis based on Sanger sequences

**Fig 2:**
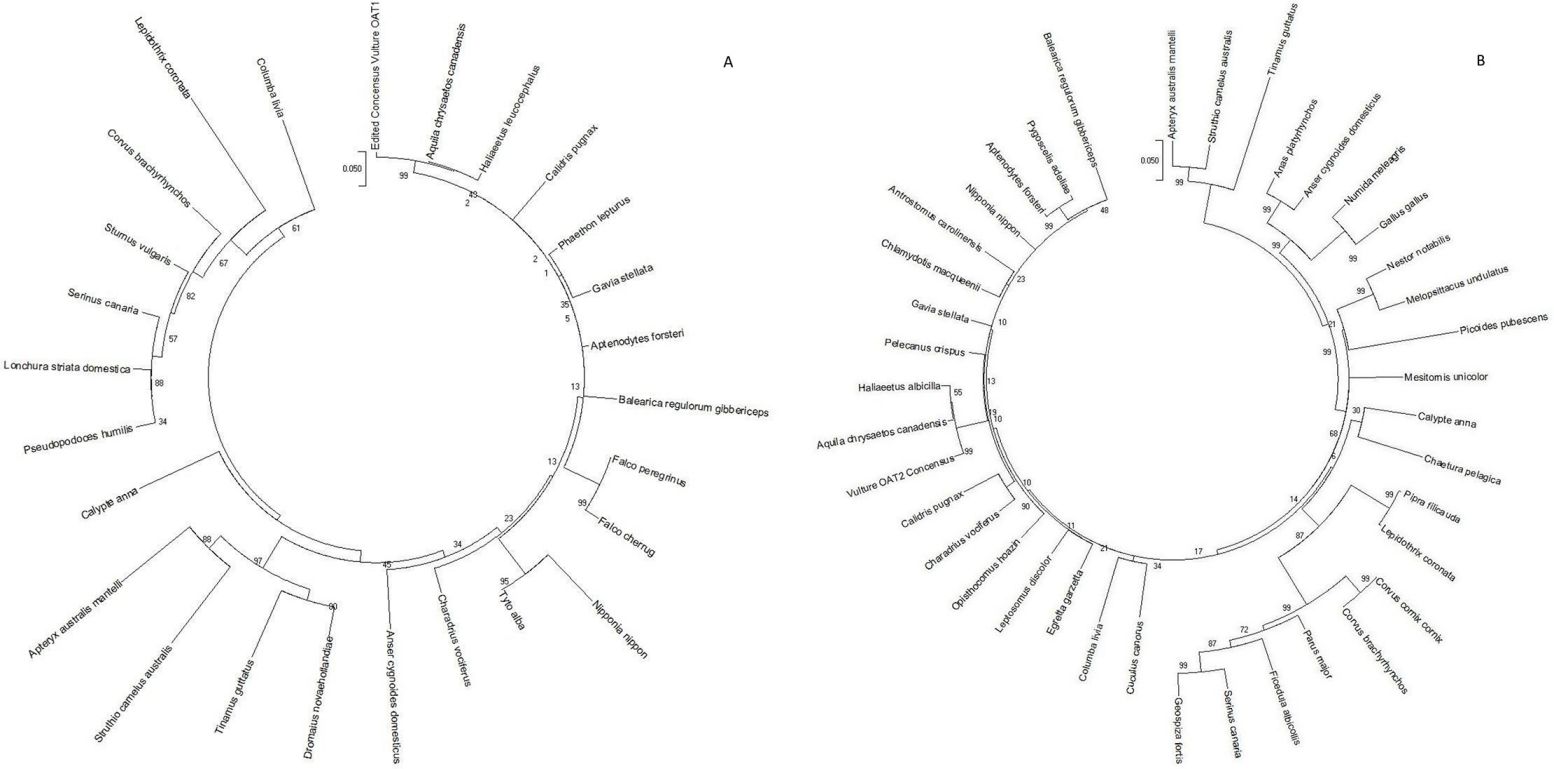
Reconstruction of phylogenetic relationships between (A) OAT1 and (B) OAT2 genes of avian species. A discrete Gamma distribution was used to model evolutionary rate differences among sites (5 categories (+G, parameter = 0.5011 and0.4500)) respectively.

The analysis involved 26 and 39 nucleotide sequences and there were a total of 1427 and 1626 positions in the final dataset for *OAT1* and *OAT2* in that order. The similarity of former species is represented by the number on the internal nodes of the branches (Fig 2). The obtained maximum likelihood trees for *OAT1* and *OAT2* revealed a close relationship between AWB vulture and eagle’s taxa while the AWB vulture and chicken did not share the same clade.

### 3.3. Protein functionality analysis and predictions

#### 3.3.1. Protein locality

**Fig 3:**
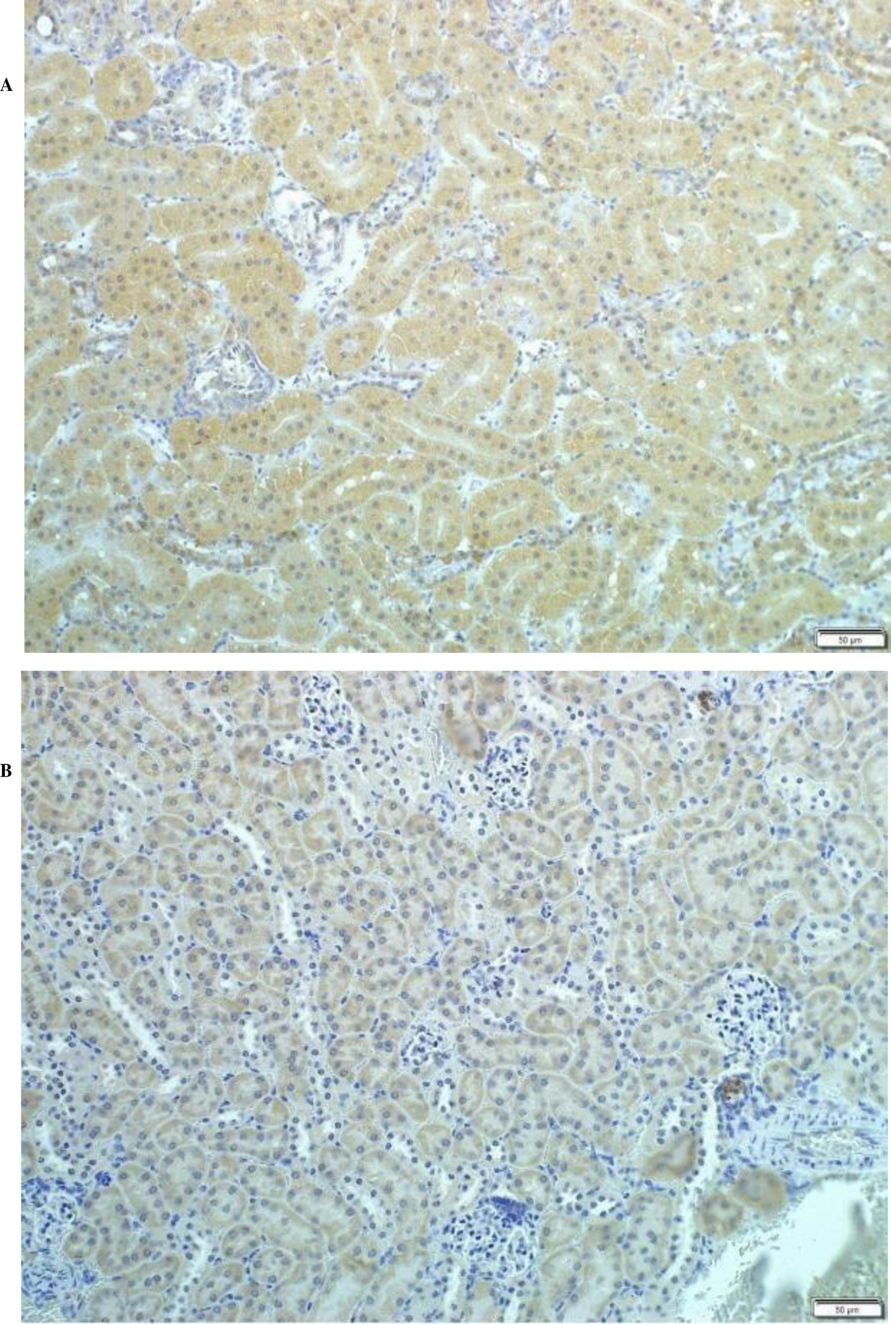
Light micrographs in 50 μm vibratome sections illustrating (A) immunostaining of OAT proteins in AWB kidneys (B) immunostaining of OAT3 protein mouse kidneys.

With rabbit *OAT3* polyclonal antibodies available this was the first step of functional analysis undertaken. With the mouse *OAT3* polyclonal antibody binding region shared 72.09% similarity with vulture *OAT1* Sanger sequence, immunohistochemistry was conducted to ascertain the distribution of *OAT1* in the AWB kidney. The mouse kidney was used as a control, as the commercial polyclonal antibodies was previously shown to be effective in the mouse. The chicken was not included in this analysis as no *OAT1* sequence was present in the NCBI database for the chicken. Light microscopy of 4 μm wax sections demonstrated immunostaining of AWB vulture`s kidney within the basolateral membrane of the proximal convoluted tubules (PCT) (Fig 3) as also observed for the mouse kidney. Some immunoreactivity was also seen on the latter kidneys on the distal convoluted tubule cells and the cortical collecting duct.

#### 3.3.2. Features of the transporter proteins

**Fig 4:**
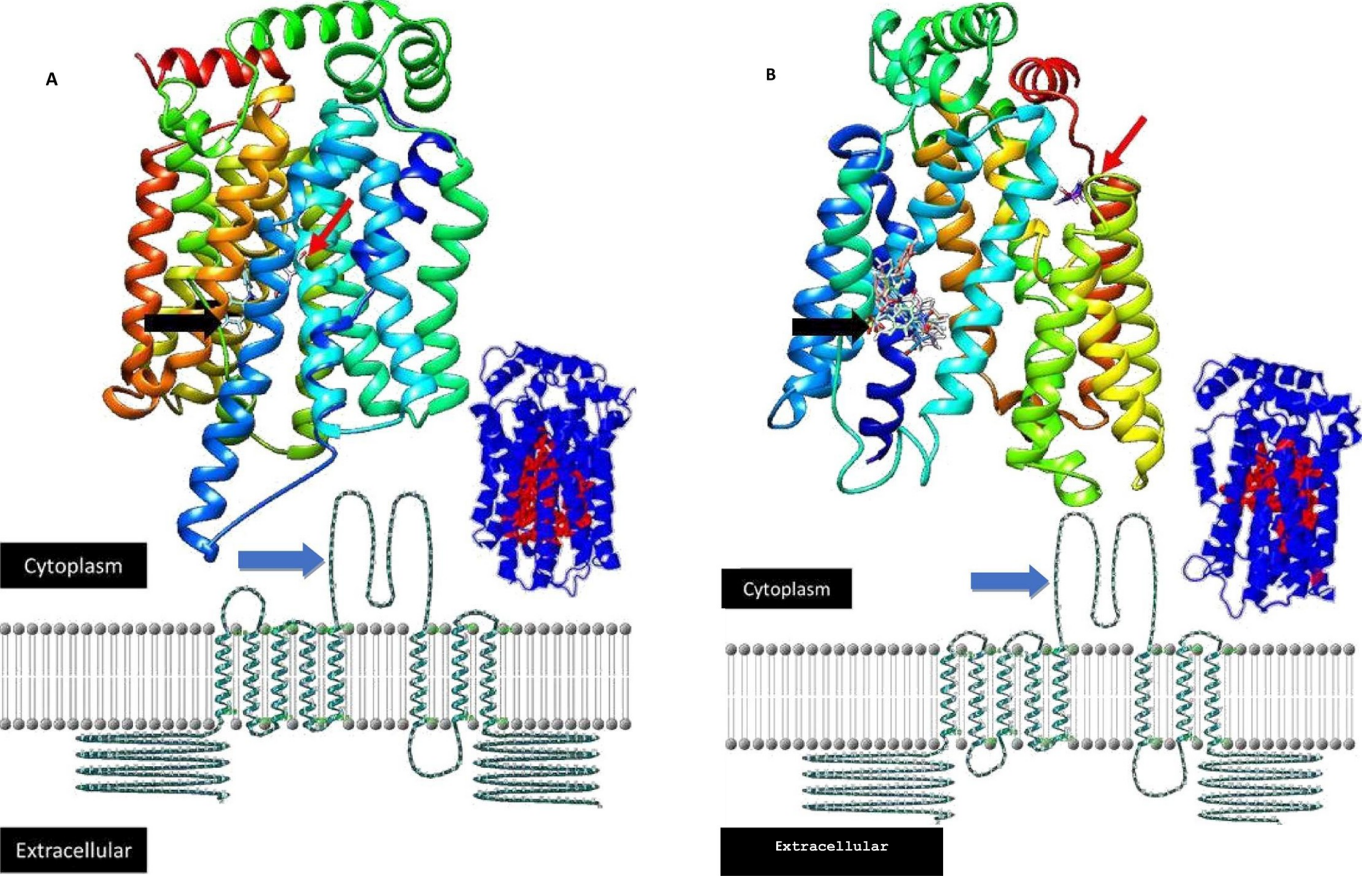
Predicted 3D and 2D organic anion transporter 2 (OAT2) from the chicken (A) and the African white-backed vulture (AWB) (B). The inserts are the identified major pocket (red). Black arrow is the predicted NSAID binding site(s), which is only diclofenac for the chicken and multiple NSAIDs for the vulture for which toxicity information is available. Red arrows are the predicted urate binding sites. Predicted Intracellular loop of chicken (C) and the AWB (D) organic anion transporter 2. Pictorial presentation of (E) AWB OAT1 representing 10 transmembrane helices and no N-glycan motif sites and the (F) AWB OAT2 representing 11 transmembrane helices and 5 N-glycan motif sites.

In subsequent protein predictive analysis, the Expasy (protparam) tool predicted that AWB vulture *OAT1* protein was made up of 449 amino acids with molecular weight of 44748.10 while the AWB vulture *OAT2* consisted of 553 amino acids with molecular weight of 57281.49. Domain analysis with scan Prosite and in Phyre2 predicted these proteins to membrane bound transport proteins that are part of the major facilitator superfamily (or solute carrier family) which transports sugars and other substrates across the cell membrane. For Phyre2, the domain analysis against similar protein was reported at a confidence of 100%. Functional prediction with TrSSP revealed that both proteins to be functional transporter of anions. PROTTER sequence database used for prediction of *N-*glycosylation sites revealed that *OAT1* had 10 putative transmembrane helices and no *N*-glycosylation sites for both the golden eagle and AWB vulture. *OAT2* had 11 transmembrane helices comprised of five *N*- glycosylation sites found at positions 31, 66, 73, 294, 330 for AWB vulture while the golden eagle had 12 transmembrane helices as well as 5 *N*-glycosylation sites at 68, 103, 110, 331, 3367. Transmembrane topology predicted that the AWB vulture *OAT1* had the presence of both an intra and extracellular loops while human *OAT1* comprises of only one intracellular loop. Vulture *OAT2* comprised of one intracellular loop as for the chicken *OAT2* (Fig 4).

### 3.4. AWB and chicken *OAT2* expression using reverse transcriptase real time PCR (RT-qPCR)

*OAT2* expression levels were determined from AWB and chicken`s renal sample. There was a slight difference in *OAT2* amplification peaks between chicken and AWB vulture expressed at cycle 20.53 and 22.17 respectively. To eliminate biasness a melting curve following RT-qPCR of housekeeping gene (GAPDH) was also evaluated for both species. The melting curve results for *OAT2* of both birds confirmed that a slight difference was present. The normalised *OAT2* expression levels for chicken and AWB vulture was observed at quantification cycles (cq) of 6.609 and 6.947 respectively.

### 3.5. Docking analysis

For final analysis, we attempted to predict how diclofenac’s would interact with the *OAT2* transporter assuming no change in protein structure on binding. One large pocket was predicted to be present for each transporter. The binding free energy predicted that the likely strongest binding sites for diclofenac and uric acid were at different points. For uric acid this was ΔG of -7.4 kcal/mol for both the vulture and chicken and -6.2 kcal/mol for humans. In contrast the ΔG was -8.6, -6.8 and -7.7 kcal/mol for diclofenac respectively for the vulture, chicken and human. By assuming that the diclofenac binding is at a fixed site, when uric acid overlapped with this specific site the ΔG for uric acid in the vulture and chicken was -5.5 and -5.7 kcal/mol respectively. No overlapping site were present for humans. When compared to other NSAIDs; meloxicam, ketoprofen, carprofen, nimesulide, and tolfenamic acid shared a binding site with a ΔGs of -7.53, -8.32, -7.47, -8.24 and -7.7 kcal/mol (Fig 4). This interesting finding shows however require further evaluation.

## 4. Discussion

With diclofenac toxicity in vultures being associated with significant increase in plasma uric acid concentration, the following study focused on identifying the organic anionic transporters present in the vulture kidney using molecular biology techniques and starting with information available for the chicken and mammals. In an unexpected finding, while a partial *OAT3* transcript was successfully identified in the chicken, as described by Dudas et al. (2005) [15], no amplification was achieved for the vulture. Further, blast analysis only demonstrated similarity of the partial chicken transcript to the *OAT1* genes of other avian species and not the chicken transcriptome. This led to the conclusion that the *OAT3*-like partial sequence previously identified in chickens was probably misclassified and that the study likely identified *OAT1*. Also as far as we could ascertain, *OAT3* have not been identified in birds in any other studies. It is also unknown why the *OAT1* partial sequences identified for the chicken was not present in the NCBI database, nor why *OAT1* is not reported to occur in the chicken. This is likely an indication that *OAT2* is the only functionally expressed in the chicken. *OAT2* has been found to be variably expressed and has also been identified as highly expressed in the mouse.

To further evaluate the OATs present in the vulture kidney, next generation sequencing and Trinity assembly revealed the presence of *OAT1* with 1468bp and *OAT2* with 2300bp genes, both with greater than 98% similar to the Golden eagle. This was subsequently confirmed by Sanger sequencing as 1350bp and 1662bp for the *OAT1* and *OAT2* transporters respectively, with up to 99% similarities to the NGS assembled sequences. The evident difference was likely a result of different birds being used for the analysis. Subsequent phylogenetic analysis showed a major diversity in the OAT transporters described thus far in various bird species, which may be attributable to evolution, mutation; gender and environment. The diversity in the sequence would likely also explain why the chicken primer of [15] didn’t amplify vulture`s *OAT3/OAT1* gene. Nonetheless the OAT transporter was more closely conserved in the family Accipitrid (vultures and eagle clade) which is not surprising due to similarity in diet and sensitivity to diclofenac as seen with the stepp eagle (*Aquila nipalensis*). Thus far under natural conditions, the stepp eagles are the only non-vulture species found to also be sensitive to effects of diclofenac [36]. In their study Sharma et al. (2014) [36] concluded that diclofenac could be toxic to other Accipitrid raptors. From this result it might be argued the phylogeny of *OAT1-2* genes in avian species may be useful in building a predictive model if the *OAT1* and *OAT2* genes can be sequenced in species of concern. While further evaluation would be required, if such a relation does exist it will be of tremendous benefit in future toxicity studies as a non-endangered surrogate species could accelerate current studies aimed evaluating the toxicity of other NSAIDs.

Next to understand the functionality of the identified OAT proteins, a number of evaluations were undertaken. Where possible comparison was made to the chicken since the species is the best studied avian in terms of uric acid excretion, and also for a yet to be explained reason as a species is also susceptible to the toxic effect of diclofenac. For the first of these evaluations, the location of the OAT transporter was determined with immunohistochemistry. For this we used commercial *OAT3* polyclonal antibodies described for the mouse, since the antibody available showed good overlapped with the vulture *OAT1* sequence of *72%*. Furthermore a polyclonal was used as it was more likely to bind to the non-target species. Following staining, the distribution of the *OAT1* transporter was localised to the proximal convoluted tubule. This result aligns with previous studies indicating that *OAT1* is localised to basolateral membrane of the proximal convoluted tubular cells in other species [17, 37–39]. While no *OAT2* antibodies were present for a similar evaluation, we have no reason to believe that its distribution would be different to *OAT1* since the two protein are expressed together by the same cell in other species [7, 11, 12].

For the next step, we ascertained if the transporter would be effective in their area of expression through *in silico* modelling. While modelling showed both *OAT1* and *OAT2* to be transporter proteins, we believe that only *OAT2* to be an anion transporter i.e. *OAT1* may not be a functional transporter. We based our conclusion on the absence of gycosylation sites for *OAT1*, which was present for *OAT2*. Studies by Tanaka et al. (2004) [40], showed that while glycosylation did not interfere with the functionality of the protein, it did play an important role in the attachment of the protein onto the plasma membrane i.e these proteins are normally within intracellular vesicles and need to be moved/translocate to the cell membrane to become functional which is unlikely in the absence of glycosylation sites. In their studies, Tanaka et al. (2004) and Zhou et al. (2005) [40, 41] were able to demonstrate that the removal of all glycosylation sites resulted in the transporters (Human *OAT1* and *OAT4*) being trapped in an intracellular compartment where they became ineffective due their location. Using this finding it is likely that *OAT1* is inactive as a transporter in the vulture or even more likely that the predicted sequence for the eagle is not truly an *OAT1* transporter. The latter may also explain why in silico analysis with TrSSP predicted no anionic activity for this protein and possibly why *OAT1* is yet to be identified in the chicken. However to strengthen this finding, studies on the transport of organic anions in the presence of specific inhibitors of the individual OAT transport proteins should be undertaken using in vitro cloning assays.

With *OAT2* deemed to be the functional protein, the last step was to determine the level of expression of the protein in comparison to the chicken. While RT-qPCR was able to confirm that *OAT2* was expressed to a similar level in both the chicken and vulture, the level of expression when corrected by the housekeeping gene reveals that the expression between the species differed by 1.3 fold, which is likely a significant finding. When comparing the vulture and chicken both species are sensitive to the toxic effects of a single dose of diclofenac. However the chicken is less sensitive with a reported LD50 (Median lethal dose) circa 10 mg/kg while the LD50 for AWB was circa 0.8 mg/kg [1, 42–44]. The slight difference in expression could be a defining feature for the differences in species susceptibility to toxicity. Moreover the 12-fold difference in LD50 may also be explained with respect to differences in amino acid sequence of the OAT as well as variable interaction of the diclofenac with OAT2 in both the species. As a next step, it would be interesting to compare *OAT2* expression of the vulture to a species that is poorly susceptible to the toxic effect of diclofenac like the duck (*Anas platyrhynchos*) or pigeon (*Columbidae*) [45].

## 5. Conclusion

In this study we conclude that AWB vulture kidney expresses *OAT1* and *OAT2* mRNA but not *OAT3*. Further with insilico modelling predicting that OAT1 protein lacks glycosylation sites, raises doubts as to whether *OAT1* is effective as a transporter. This is highly suggestive that *OAT2* is likely the main uric acid transporter in AWB vulture. Further with expression studies indicating slight differences in *OAT2* expression levels between the chicken and vulture, this may explain the marginal differences in sensitivity to diclofenac between these two species. In order to strengthen the results above, other studies should be conducted to reinforce where *OAT2* transporter is functional on the basolateral membrane.

## Supporting information

S1 Table Avian OAT1 and OAT2 sequences used for phylogenetic analysis. (DOCX)
