# Supplementary material for: Molecular characterization of Gyps africanus (African white-backed vulture) organic anion transporter 1 and 2 expressed in the kidney
Source: PLoS One. 2021 May 4;16(5):e0250408. doi: 10.1371/journal.pone.0250408 (PMC8096082; doi:10.1371/journal.pone.0250408)
Supplement: S1 Table — (DOCX) [file pone.0250408.s001.docx]

**Molecular characterization of *Gyps africanus* (African white-backed vulture) Organic Anion Transporter 1 and 2 expressed in the kidney.**

Bono Nethathe^1, 4^, Rephima Phaswane^2^, Aron Abera^3^, Vinny Naidoo^1^

S1 Table : Avian OAT1 and OAT2 sequences used for phylogenetic analysis.

| Species | Common name | OAT1 | OAT2 |
| --- | --- | --- | --- |
| Anas platyrhynchos | Mallard duck |  | XM_027453544.1 |
| Anser cygnoides domesticus | Domestic goose | XM_013202223.1 | XM_013194917.1 |
| Aptenodytes forsteri | Emperor penguin | XM_019470643.1 | XM_009272605.1 |
| Aquila chrysaetos canadensis | Golden eagle | XM_011601043.1 | XM_011585794.1 |
| Balearica regulorum gibbericeps | East African grey crowned crane | XM_010301204.1 | XM_010301419.1 |
| Calidris pugnax | Ruff | XM_014966557.1 | XM_014962397.1 |
| Calypte anna | Anna`s hummingbird | XM_008496106.1 | XM_008504985.1 |
| Caprimulgus carolinensis | Chuck-will`s widow | XM_010173171.1 | XM_010168880.1 |
| Charadrius vociferus | Killdeer | XM_009883135.1 | XM_009894862.1 |
| Columba livia | Rock pigeon | XM_021301243.1 | XM_005512038.2 |
| Corvus brachyrhynchos | American crow | XM_017747256.1 | XM_008635764.2 |
| Falco cherrug | Saker falcon | XM_027811268.1 |  |
| Falco peregrinus | Peregrine falcon | XM_027792445.1 |  |
| Fulmarus glacialis | Northern fulmar | XM_009583211.1 | XM_009587322.1 |
| Gallus gallus | Chicken |  | NM_001199438.1 |
| Gavia stellata | Red throated loon | XM_009814693.1 | XM_009815441.1 |
| Geospiza fortis | Medium ground-finch | XM_014311357.1 | XM_005422609.1 |
| Haliaeetus leucocephalus | Bald eagle | XM_010570679.1 |  |
| Lepidothrix coronate | Blue-crowned manakin | XM_017840174.1 | XM_017818790.1 |
| Lonchura striata domestica | Society Finch | XM_021545734.1 |  |
| Nipponia nippon | Crested ibis | XM_009460680.1 | XM_009468586.1 |
| Opisthocomus hoazin | Hoatzin | XM_009937221.1 | XM_009939956.1 |
| Pelecanus crispus | Dalmatian pelican | XM_009489468.1 | XM_009478709.1 |
| Phaethon lepturus | White-tailed trophic bird | XM_010292254.1 | XM_010285480.1 |
| Pseudopodoces humilis | Tibetan ground tit | XM_005533638.1 |  |
| Serinus canaria | Common canary | XM_018914013.1 | XM_009096157.2 |
| Struthio camelus australis | South African ostrich | XM_009671879.1 | XM_009673139.1 |
| Sturnus vulgaris | Common starling | XM_014893033.1 | XM_014891915.1 |
| Tinamus guttatus | White- throated tinamou | XM_010227345.1 | XM_010217054.1 |
| Tyto alba | Barn Owl | XM_009972042.1 |  |
| Apteryx australis mantelli | North island brown kiwi | XM_013942757.1 | XM_013954347.1 |
| Dromaius novaehollandiae | Emus | XM_026122546.1 |  |
| Chaetura pelagica | Chimney swift |  | XM_010005012.1 |
| Chlamydotis macqueenii | MacQueen bustard |  | XM_010123992.1 |
| Corvus cornix cornix | Hooded crow |  | XM_010411086.2 |
| Egretta garzetta | Little egret |  | XM_009641620.1 |
| Eurypyga helias | Sunbittern |  | XM_010161130.1 |
| Ficedula albicollis | Collard flycatcher |  | XM_005042781.2 |
| Haliaeetus albicilla | White-tailed eagle |  | XM_009922646.1 |
| Leptosomus discolor | Cuckoo roller |  | XM_009961411.1 |
| Meleagris gallopavo | Turkey |  | XM_003203777.3 |
| Melopsittacus undulatus | Budgerigar |  | XM_005152033.2 |
| Mesitornis unicolor | Brown roatelo |  | XM_010182673.1 |
| Nestor notabilis | Kea |  | XM_010016946.1 |
| Numida meleagris | Helmeted guineafowl |  | XM_021389777.1 |
| Parus major | Great tit |  | XM_015620291.2 |
| Phalacrocorax carbo | Great cormorant |  | XM_009502511.1 |
| Picoides pubescens | Downy woodpecker |  | XM_009903833.1 |
| Pterocles gutturalis | Yellow-throated sandgrouse |  | XM_010077045.1 |
| Pygoscelis adeliae | Adélie penguin |  | XM_009331354.1 |
| Tauraco erythrolophus | Red-crested turaco |  | XM_009990757.1 |
| Zonotrichia albicollis | White-throated sparrow |  | XM_005486767.1 |
